# Process development of sugar beet enzymatic hydrolysis with enzyme recycling for soluble sugar production

**DOI:** 10.1007/s00449-022-02742-8

**Published:** 2022-07-02

**Authors:** Yike Chen, Natthiporn Aramrueang, Steve Zicari, Ruihong Zhang

**Affiliations:** 1 Department of Biological and Agricultural Engineering, University of California, Davis, USA; 2 Department of Biotechnology, Faculty of Science, Mahidol University, Bangkok, Thailand

**Keywords:** Enzyme recycling, Enzymatic hydrolysis, Sugar beet, Membrane filtration, Process development

## Abstract

Enzymatic hydrolysis of sugar beets for achieving liquefaction and sugar release is a critical step for beet-ethanol production. An enzyme recycling process was developed in this study to reduce the economic uncertainty raised by the high costs of enzymes by reducing the fresh enzyme usage. A mixture of cellulases and pectinases was used in the beet hydrolysis. The hydrolysate was centrifuged and then processed through a 50 kDa molecular weight cut-off polyethersulfone membrane to recover enzymes from the liquid. Liquid enzyme recycling with 50% fresh enzyme addition achieved a similar liquefaction extent and sugar yield compared to the positive control with 100% fresh enzyme. Solid enzyme recycling showed a lower liquefaction efficiency, requiring at least 75% of fresh enzyme addition for a comparable liquefaction extent. Five sequential batches of hydrolysis with liquid enzyme recycling were successfully conducted to hydrolyze sugar beets with similar liquefaction extents and sugar yields.

## Introduction

Sugar beet is used widely for sugar production worldwide. Most of the previous research on biofuel production has been focused on utilization of the byproducts of beet refineries, such as beet pulp, thick juice, and molasses as feedstock [1–4]. It was estimated that around 160 kg of sugar, 500 kg of wet pulp, and 38 kg of molasses could be generated from 1 metric ton (MT) of raw sugar beet [5]. Recent studies have also examined ethanol production using the whole sugar beets [6, 7].

Sugar beet is a promising feedstock for bioethanol production, especially in California, due to its high sugar yield and potential for year-round harvesting. Batch enzymatic hydrolysis of sugar beets for bioethanol production has been developed at laboratory scale and successfully demonstrated in pilot plant studies at University of California, Davis [6]. Beet hydrolysis with enzymes is one of the key processes used in this conversion system, liberating the sucrose and water inside the beet cells. In addition, other structural carbohydrates contained in beet cell walls, such as cellulose, hemicellulose and pectin, can also be hydrolyzed by the enzymes for producing additional soluble sugars. The ethanol yield with enzymatic hydrolysis could potentially increase from 75% to over 90% of the theoretical conversion of total soluble six-carbon (C6) sugars [8]. Moreover, a significant amount of water and energy could be saved by applying the enzymatic hydrolysis process compared to the conventional hot water extraction method. However, enzymes are a high-cost input in the beet-to-ethanol conversion system. Economic feasibility of ethanol production from sugar beets is very sensitive to the enzyme costs. The estimated enzyme cost was $0.12/L ethanol, which represents more than 10% of the operating cost [6].

Enzyme recovery from the hydrolysate and reuse in the subsequent batch of hydrolysis will reduce the use of fresh enzymes. Jørgensen and Pinelo [9] reviewed different enzyme recycling strategies in lignocellulosic biorefineries, as well as their industrial applications. Liquid recycling is a straightforward method to recycle the soluble enzymes. Solid–liquid separation and membrane filtration have been widely used in various industrial applications. Previous research showed that about 80% of the original hydrolysis yield could be kept after four sequential batches of hydrolysis of ethanol pre-treated Lodgepole pine with liquid recycling [10]. On the other hand, some of the enzymes are expected to bind to the solids after the hydrolysis of lignocellulosic materials. Weiss et al. [11] studied solids recycling during cellulase hydrolysis of dilute acid pre-treated corn stover and found that by recycling 50% of the separated solids and adding 67% of the fresh enzymes, the subsequent batches of hydrolysis could reach a 60% hydrolysis yield for at least four cycles. Enzymatic hydrolysis together with enzyme recycling has a potential to be integrated into a sugar beet refinery to reduce the enzyme loadings, which could eventually make the hydrolysis process more economically feasible at industrial scale. The feasibility of enzyme recycling during enzymatic hydrolysis of sugar beets was investigated in this study with a goal to develop an enzyme recycling process to recover and reuse the enzymes. The objectives were to (1) evaluate the efficiency of separation methods for enzymes recycle; (2) develop enzyme recycling process and investigate the performance of recycled enzyme; and (3) study sequential batch enzymatic hydrolysis of sugar beets.

## Materials and methods

### Feedstock preparation

Sugar beets (*Beta vulgaris, L*) were collected from research plots at the University of California field station in the Imperial Valley, California. Whole sugar beet roots were chopped and grounded by a food processor (Cuisinart, USA) and stored in bags at − 20 °C for later enzymatic hydrolysis experiments. Sugar beet samples were analyzed for moisture content (MC), total solids (TS), volatile solids (VS) and ash contents using the standard methods described in the *Standard Methods for the Examination of Water and Waste Water* [12]. Prior to each experiment, sugar beet samples were thawed, and TS was measured again by the HB43-S Halogen Moisture Analyzer (Mettler Toledo, USA) to check the sample quality, as well as calculate the total solids and enzyme loadings.

### Enzymatic hydrolysis of sugar beets

Enzymatic hydrolysis was performed by mixing the ground sugar beets, enzyme mixture and 0.05 M sodium citrate buffer (pH 4.8). The enzymes included commercial cellulase (*Cellic CTec 2*, 125 FPU/mL) and pectinase (*NS22119,* 10,007 PGU/mL) products provided by Novozymes North America, Inc. (Franklinton, NC). The enzyme loadings were 35 and 25 mL/kg TS of sugar beet for cellulase and pectinase, respectively, which were previously determined as optimum levels by Zicari [6].

The first batch of enzymatic hydrolysis was carried out in 500 mL bottles at 50 °C with a working volume of 150 mL at 20% TS loading. The beets and enzymes were continuously mixed using a Roll-A-Cell roller bottle reactor (New Brunswick Scientific, USA). The hydrolysate was used for the enzyme separation and recovery experiment, as well as the subsequent batch of hydrolysis with different proportions of recycled and fresh enzymes. The subsequent batch of hydrolysis was conducted in 50 mL Falcon tubes with a working volume of 15 mL at 20% TS loading. An Innova 4000 incubator shaker (New Brunswick Scientific, USA) was used to provide mixing and maintain the temperature at 50 °C. Two sample tubes were sacrificed each time for measurements of liquefaction, soluble sugar and protein concentrations, and enzyme activity.

### Analytical methods

Liquefaction was calculated by dividing the mass of supernatant decanted after centrifugation by the initial total mass as shown in Eq. 1.

1 $$\mathrm{Liquefaction} \, \left(\mathrm{\%}\right)=\frac{\mathrm{mass \, of \, supernatant \, decanted \, after \, centrifugation }\left(\mathrm{g}\right)}{\mathrm{initial \, total \, mass } \, \left(\mathrm{g}\right)}\times 100\mathrm{\%}.$$

Soluble sugar concentration was measured by a YSI 2700 biochemistry analyzer (Xylem, USA) equipped with dual glucose and sucrose channels. 5 g/L sucrose and 2.5 g/L glucose standard solutions were run as external standards for the analyzer. Glucose concentration was then converted to its equivalent sucrose concentration by assuming all glucose was generated by sucrose hydrolysis. Total soluble sugar concentration in the supernatant was reported as the total sucrose concentration in the unit of grams of sucrose-equivalent per liter (g suc eq/L). Soluble protein concentration measurements were conducted using the Bio-Rad standard protein assay adapted from the protein–dye binding method developed by Bradford [13].

Enzyme activity measurements were found to be challenging after the hydrolysis because the enzymes could be inhibited by the high sugar concentration. Errors might also be introduced if the samples were diluted too much to eliminate the effect of product inhibition. Separating enzymes and soluble sugars, thus, became necessary to the enzyme activity measurements. Centrifugal ultrafiltration tubes with a 50 kDa MWCO PES membrane were used to retain enzymes in the retentate and wash out the soluble sugars at the same time. Washing twice with citric acid buffer solution was experimentally determined to be effective to eliminate the sugar inhibition and preserve most of the soluble protein after enzymatic hydrolysis for conducting enzyme activity assays. Enzyme activities in the supernatant were measured to investigate the amount of fresh enzymes needed for a subsequent batch of hydrolysis. Cellulase and pectinase activities were measured by the filter paper and polygalacturonic acid assays [14, 15] after washing the supernatant three times by citrate buffer using 50 mL Corning ultrafiltration concentrators (Sigma-Aldrich, USA) to eliminate the product inhibition. The units of cellulase and pectinase activities were reported as filter paper unit (FPU) and polygalacturonase unit (PGU), respectively. The initial enzyme activities were estimated by multiplying the enzyme activities in the stock solution and the volume of the stock solution added.

### Enzyme separation and recovery

After the first batch of enzymatic hydrolysis, the hydrolysate was centrifuged at 5000×*g* for 30 min to separate the supernatant from the residual solids. The composite supernatant was further subjected to the ultrafiltration to separate and concentrate the enzymes in the retentate. The permeate stream, with mostly soluble sugars, was collected and saved as the main product.

The ultrafiltration was conducted using the SEPA Cell membrane filtration unit (Sterlitech, USA), which is a bench scale cross flow filtration unit. The SEPA Cell is equipped with a feed pump (110 V, 60 Hz), aluminum cell holder, membrane cell with the flat sheet membrane, hydraulic hand pump, and outlet control valve. The membrane active area of the SEPA Cell is 140 cm^2^ (24 in^2^). The transmembrane pressure was controlled at 8 bar (116 psi) by adjusting the outlet valve. The flux of the permeate, and soluble sugar and protein concentrations in the permeate with two different membrane sizes were tested. The polyethersulfone (PES) flat sheet membranes were provided by Synder Filtration (Vacaville, CA) with 10 and 50 kDa molecular weight cut-off (MWCO). The ultrafiltration unit was operated at room temperature and 8 bar (116 psi) outlet pressure.

The accumulated volume of the permeate was measured at different time points to calculate the flux (*F*) using Eq. 2:

2 $$F (L/h {m}^{2})=\Delta V/(\Delta t\times A),$$

where, $\Delta V$ is the volume of the permeate accumulated between two measurements (*L*), $\Delta t$ is the time difference between two measurements (*h*), and *A* is the membrane active area (m^2^).

The removal efficiencies or rejection rates (*R*) of soluble protein and sugar were calculated by Eq. 3:

3 $$R(\% ) = \left( {1 - \frac{{C_{{\text{p}}} }}{{C_{{\text{S}}} }}} \right) \times 100,$$

where, ${C}_{\mathrm{p}}$ and ${C}_{\mathrm{S}}$ represent the soluble protein or sugar concentrations in the permeate and supernatant (g/L), respectively.

### Enzymatic hydrolysis with enzyme recycling

#### Liquid recycling

The hydrolysate after a batch of enzymatic hydrolysis of sugar beets with fresh enzymes was subject to an enzyme recycling process, including centrifugation and ultrafiltration. Retentate with recycled enzymes was added back to a subsequent batch of hydrolysis with different amounts of additional fresh enzymes. The design criteria were to achieve an 80% liquefaction within 8 h, as it was concluded to be both technically and economically feasible [6].

Enzymatic hydrolysis with liquid recycling was conducted in 50 mL centrifuge tubes with a 20% TS loading and 15 g total weight. 2.5 g sodium citrate buffer required for initial TS adjustment was substituted by the retentate from the last batch of hydrolysis. Different amounts of fresh enzymes were added to compensate for the enzyme losses during the separation and recovery processes. In this experiment, 25%, 50%, and 75% of the original amount of fresh enzymes were added to each treatment. No fresh enzyme addition and 100% fresh enzyme without enzyme recycling were included as the negative and positive controls, respectively. The enzymatic hydrolysis was carried out in Innova 4000 incubator shaker (New Brunswick Scientific, USA) at 50 °C. Two Falcon tubes were sacrificed at different time points for soluble sugar and protein concentrations, and liquefaction measurements as described above.

#### Solid recycling

Enzymatic hydrolysis with solid recycling was carried out in a similar way. Instead of going through ultrafiltration, solids separated from centrifugation were collected and added back to the subsequent batch of hydrolysis. To adjust to a 20% initial TS loading, 2.5 mL sodium citrate buffer was mixed with 12.5 g sugar beets to achieve a final weight of 15 g in 50 mL Falcon tubes. Additionally, 25%, 50%, and 75% of the original amount of fresh enzyme was added likewise. Negative controls with no fresh enzyme addition, and positive controls with 100% fresh enzymes, and no enzyme recycling were also included.

#### Sequential batches of enzymatic hydrolysis with enzyme recycling

Five sequential batches of hydrolysis were conducted in 50 mL Falcon tubes with the recycled retentate and 50% fresh enzyme. After each batch of hydrolysis, the hydrolysate was subjected to centrifugation at 5000×*g* for 30 min. The supernatant was then transferred into a 50 mL Corning ultrafiltration concentrator (Sigma-Aldrich, USA). The concentrator has low binding PES membranes with 50 kDa MWCO, similar to the flat sheet membrane used in the SEPA cell filtration unit. The centrifugal ultrafiltration process was conducted at 5000×*g* for 40 min to achieve a 75% volume reduction. The concentrated retentate with recycled enzymes was added back to the next batch of enzymatic hydrolysis with ground sugar beets and 50% of the original volume of the fresh enzyme mixture. All five batches of the enzymatic hydrolysis were incubated in the Innova 4000 incubator shaker (New Brunswick Scientific, USA) at 50 °C and 180 rpm for 8 h. Liquefaction and soluble sugar concentrations were measured at the end of each hydrolysis experiment. Sugar yield (${Y}_{\mathrm{sugar}}$) was calculated by Eq. 4:

4 $${Y}_{\mathrm{sugar}} (\mathrm{\%})=\frac{{C}_{\mathrm{sugar}} \times {V}_{\mathrm{supernatant}}}{{M}_{\mathrm{initial}}}\times 100,$$

where ${C}_{\mathrm{sugar}}$ is the final sugar concentration in the liquid (g sucrose eq/L), ${V}_{\mathrm{supernatant}}$ is the final supernatant volume (*L*), and ${M}_{\mathrm{initial}}$ is the initial dry mass of sugar beets (*g*).

#### Enzymatic hydrolysis with surfactant addition

Tween 80, poly(oxyethylene)_20_-sorbitanmonooleate, obtained from Sigma-Aldrich (St. Louis, MO), was added at the beginning (*t* = 0 h) and in the middle (*t* = 4 h) of the enzymatic hydrolysis as a non-ionic surfactant. Tween 80 was added into the 50 mL Falcon tubes at a loading of 0.1 g/g dry sugar beet. Negative control without surfactant was included in the experimental design. All of the other substrate, enzyme loadings, and hydrolysis conditions were kept the same as previously stated. Liquefaction and sugar concentration were measured at different time points. Enzyme activities, FPU and PGU, in the liquid phase were measured at the end after washing with the citrate buffer solution for three times.

All of the experiments were conducted in duplicate. Average values with associated error bars representing the value range of replicates are shown in the figures. The Analysis of Variance (ANOVA) was conducted using Prism (GraphPad, USA) to statistically determine the significant differences between treatments.

## Results and discussion

### Ultrafiltration with different molecular weight cut-off (MWCO) membranes

**Fig. 1:**
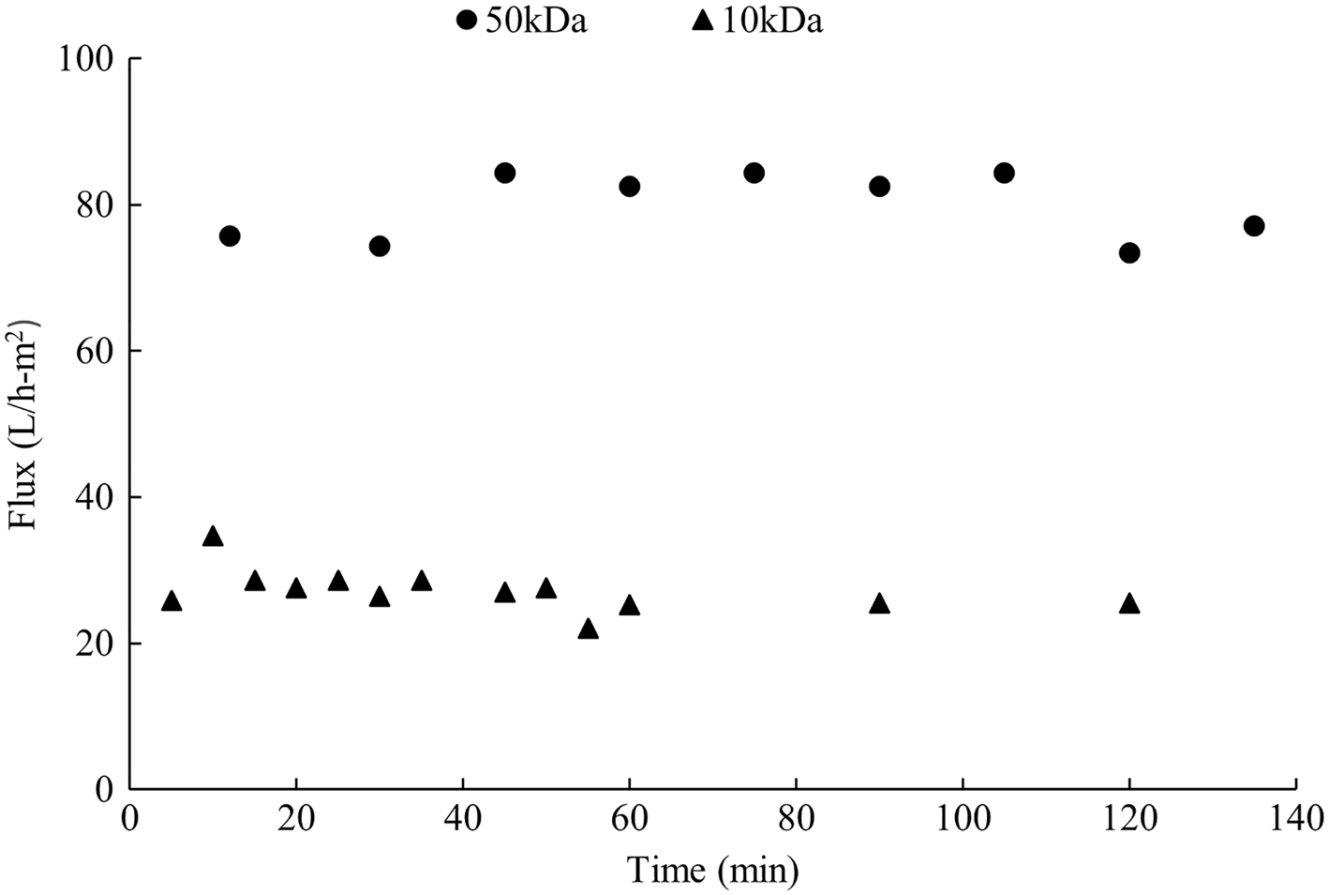
Permeate flux with different membrane sizes

The feasibility of scaling up an ultrafiltration process is subject to the flux of the product stream, product loss or damage during the process, and value of the final product [16]. In this case, the permeate stream with the soluble sugar is considered as the main product. Figure 1 shows the permeate flux with different membrane sizes.

The flux with a 50 kDa MWCO membrane was almost 3 times higher than that with a 10 kDa MWCO membrane. This could be explained by the Hagen–Poiseuille equation (Eq. 5), which gives a simple relationship between the flux (*J*) and the membrane porosity ($\varepsilon )$:

5 $$J \left(\frac{L}{{m}^{2} s}\right)=\frac{\varepsilon {r}^{2}\Delta P}{8\mu {L}_{\mathrm{m}}},$$

where $r$ is the radius of the pores (m), $\Delta P$ is the pressure drop across the membrane (Pa), $\mu$ is the viscosity of the permeate (Pa s), and ${L}_{\mathrm{m}}$ is the length of the pores in the membrane (m). However, the theoretical permeate flux is hard to achieve because a simple cylindrical pore structure is assumed in the equation while most of the pore structure is a complex tortuous channel in reality. Additionally, no significant fouling effect was observed with both membranes after 2 h of continuous operation using the bench scale membrane filtration unit.

The quality of the final product was determined by measuring the soluble sugar and protein concentrations. In this study, a high rejection rate of the hydrolytic enzymes and a low rejection rate of the soluble sugars would be preferred. Table 1 summarizes the soluble enzyme and sugar concentrations in the supernatant after centrifugation and permeate after ultrafiltration.

**Table 1:** Protein and sugar concentrations in different streams during ultrafiltration

	Protein concentration (g/L)		Sugar concentration (g suc eq./L)	
	50 kDa MWCO	10 kDa MWCO	50 kDa MWCO	10 kDa MWCO
Supernatant	0.34 ± 0.00	0.37 ± 0.01	138.63 ± 1.71	137.24 ± 3.68
Permeate	0	0	132.17 ± 2.31	129.21 ± 1.87
*R* (%)	100	100	4.66	5.85

No soluble protein was detected in the permeate with both 10 kDa and 50 kDa MWCO membranes, meaning that both membranes were able to retain all the enzymes in the retentate. This agrees with the previous research showing that most of the lignocellulosic enzymes have a molecular weight between 60 and 90 kDa [17]. Soluble sugars were evenly distributed in each stream, and no significant sugar losses during ultrafiltration were noted using either membrane (*p* = 0.0593 for 10 kDa membrane and *p* = 0.1134 for 50 kDa membrane). Therefore, both 10 and 50 kDa MWCO membranes could successfully retain all the enzymes in the retentate with little rejection of soluble sugars. Since 50 kDa MWCO membrane showed a significantly higher flux, it was used in the following enzyme recycling experiments.

### Enzymatic hydrolysis with liquid recycling

**Fig. 2:**
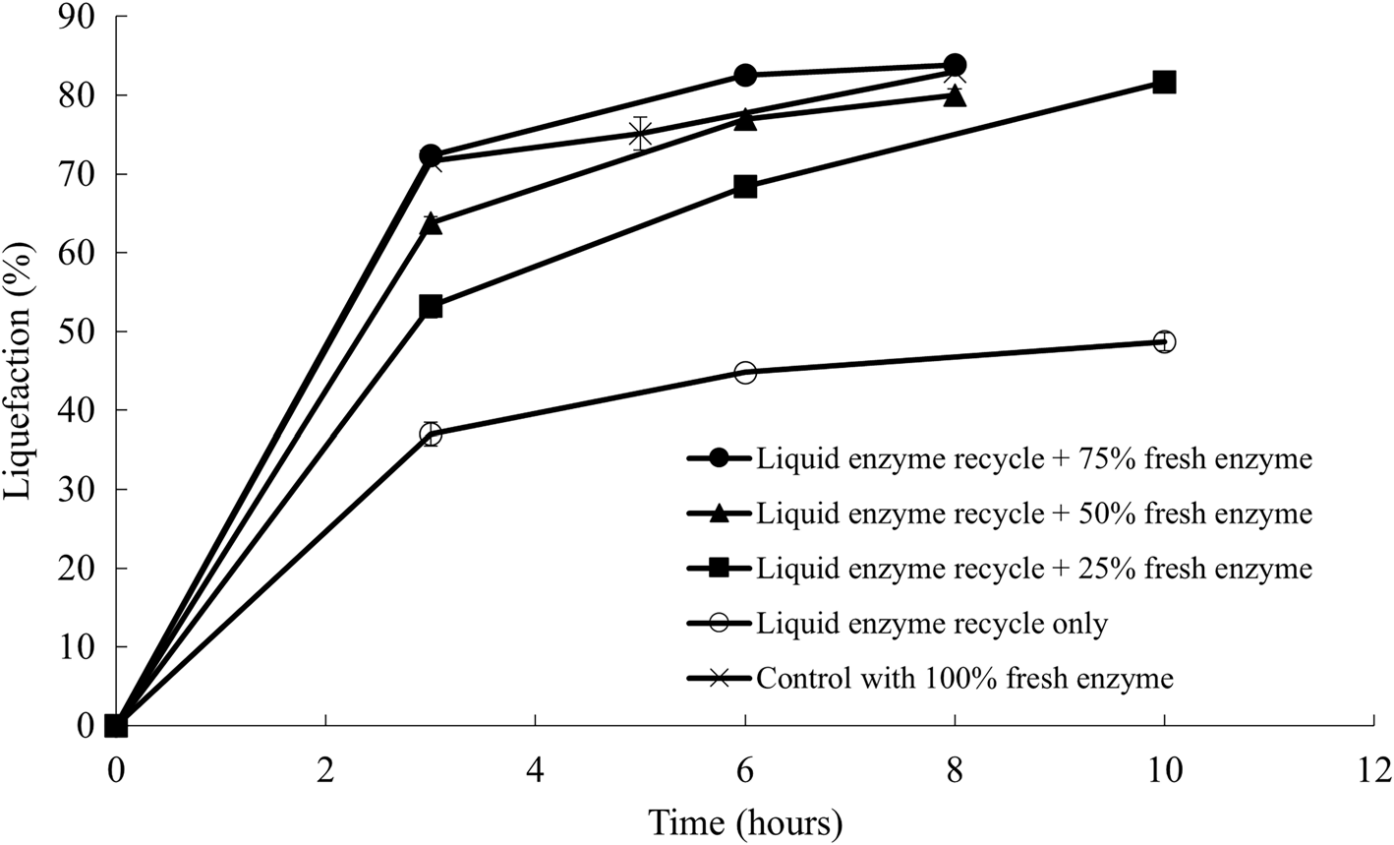
Liquefaction with recycled enzyme in the liquid hydrolysate and fresh enzyme

Liquefaction results of each treatment with respect to time were shown in Fig. 2. Negative control with only buffer was not shown in the figure since no liquefaction was observed during the 8-h hydrolysis. Treatments with recycled enzymes and 50% and 75% of original fresh enzymes addition reached approximately 80% liquefaction within 8 h. Liquid recycle with 25% fresh enzyme took 10 h to liquefy 80% of the ground sugar beets. However, enzymatic hydrolysis with only recycled enzymes reached a maximum of 50% liquefaction after 10 h. Therefore, 100% of the enzymes cannot be recycled using the liquid recycling process. Liquid recycling with additional 50% fresh enzyme was shown to be an effective way to hydrolyze sugar beets without compromising the liquefaction efficiency. In other words, 50% of the fresh enzyme could be saved for the subsequent batch of beet hydrolysis with liquid recycling.

**Fig. 3:**
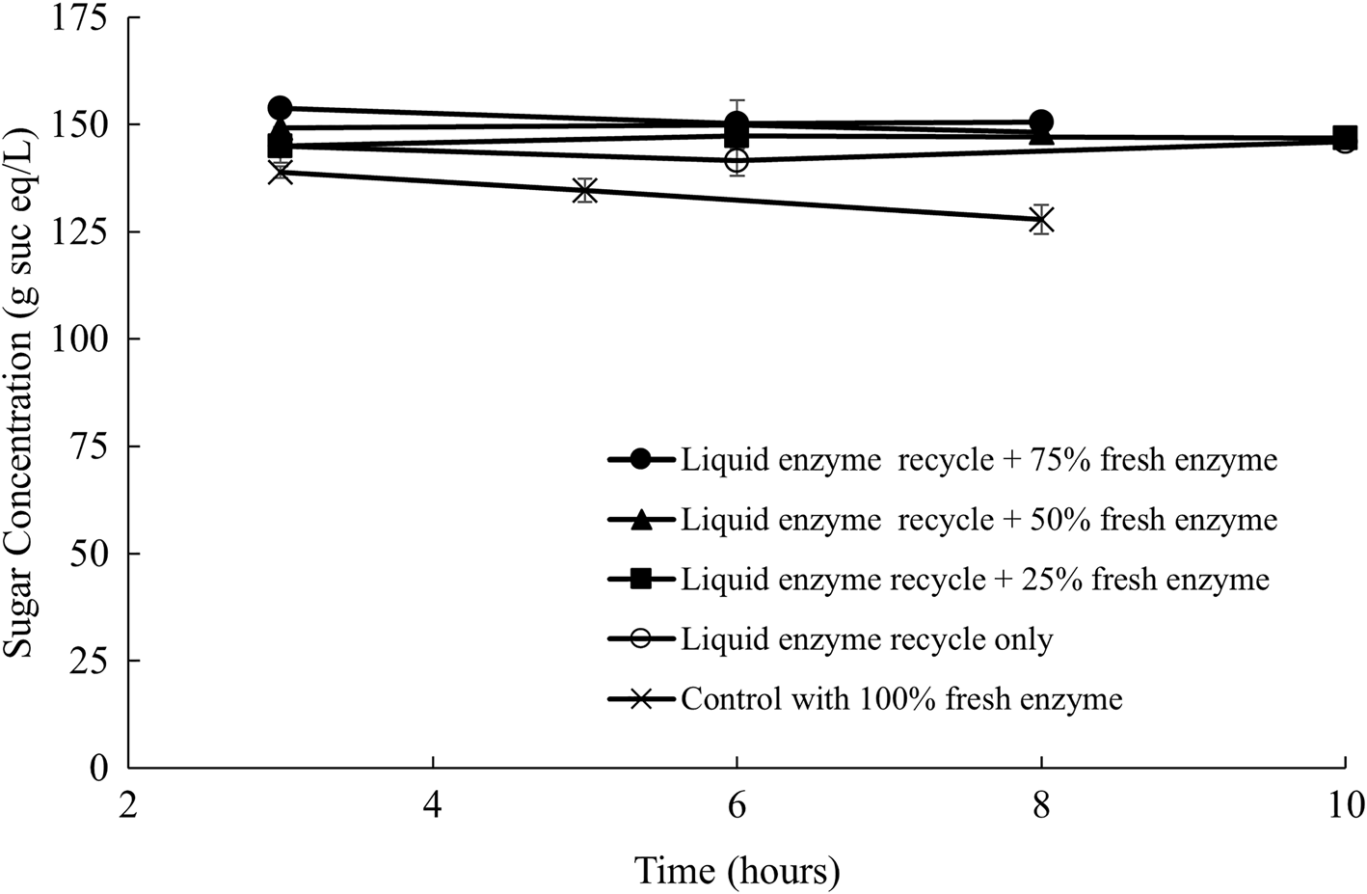
Sugar concentration in the supernatant with liquid recycling and fresh enzyme

Sugar concentration was also measured in the supernatant at different time points and depicted in Fig. 3. It was concluded that the sugar concentration stayed constant throughout the hydrolysis process. The average sugar concentration was around 148 g sucrose eq/L after 8 h, accounting for 87% of the sucrose in the sugar beet by assuming a 68% initial sucrose content on a dry basis. Since there was no significant difference in the sugar concentration over the course of the enzymatic hydrolysis, the higher liquefaction would result in a higher sugar yield. The control with 100% fresh enzyme had a lower final sugar concentration of 127 g sucrose eq/L than those with liquid recycling. It should be noted that the soluble sugars contained in the retentate were not subtracted in the treatments with liquid recycling. Mass balance showed that around 14.1% of the soluble sugars were recycled back to the subsequent batch of hydrolysis along with the enzymes by liquid recycling. The difference in sugar concentrations between the treatments with enzyme recycling and control could also be accounted by the hydrolysis of residual oligosaccharides contained in the retentate.

**Fig. 4:**
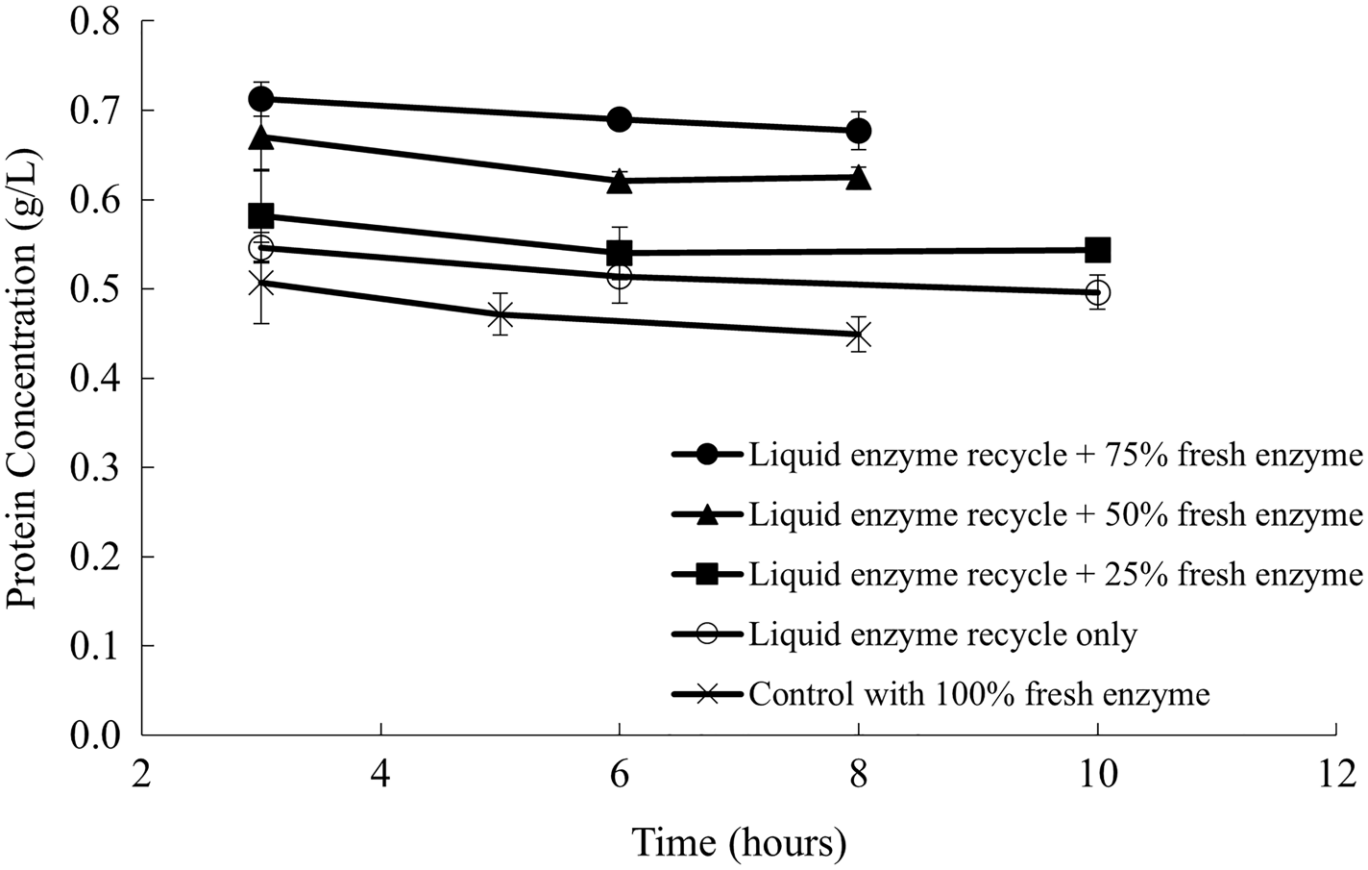
Protein concentration in the supernatant with liquid recycling and fresh enzyme

Similar to the sugar concentration, the protein concentration in the supernatant stayed constant during the entire hydrolysis process (Fig. 4). The control with 100% fresh enzyme had the lowest protein concentration in the supernatant. Liquid recycling with a higher amount of fresh enzyme addition had a higher soluble protein concentration. Since the volume of the liquid added to the subsequent batch of hydrolysis was consistent, the difference in the soluble protein concentration in the supernatant could be explained by the different volumes of fresh enzyme addition. On the other hand, soluble protein concentration measurements did not explain the difference in the liquefaction with different proportions of fresh enzyme addition. One of the greatest limitations of the Bradford protein concentration measurement is that the dye binds not only the active enzymes, but also the inactive enzymes and soluble protein from the beets. For instance, the protein concentration of liquid recycling with no additional fresh enzyme was statistically the same as that of the positive control. But the positive control had a much higher liquefaction rate and extent than liquid recycling without fresh enzyme addition. Experiments with fluorescence labeled enzymes is a possible approach to help differentiate soluble proteins from sugar beet proteins or added enzymes. This strategy could also aid in displaying the distribution of enzymes in the liquid or solid phase.

### Enzyme activity measurement during enzyme recycling

**Fig. 5:**
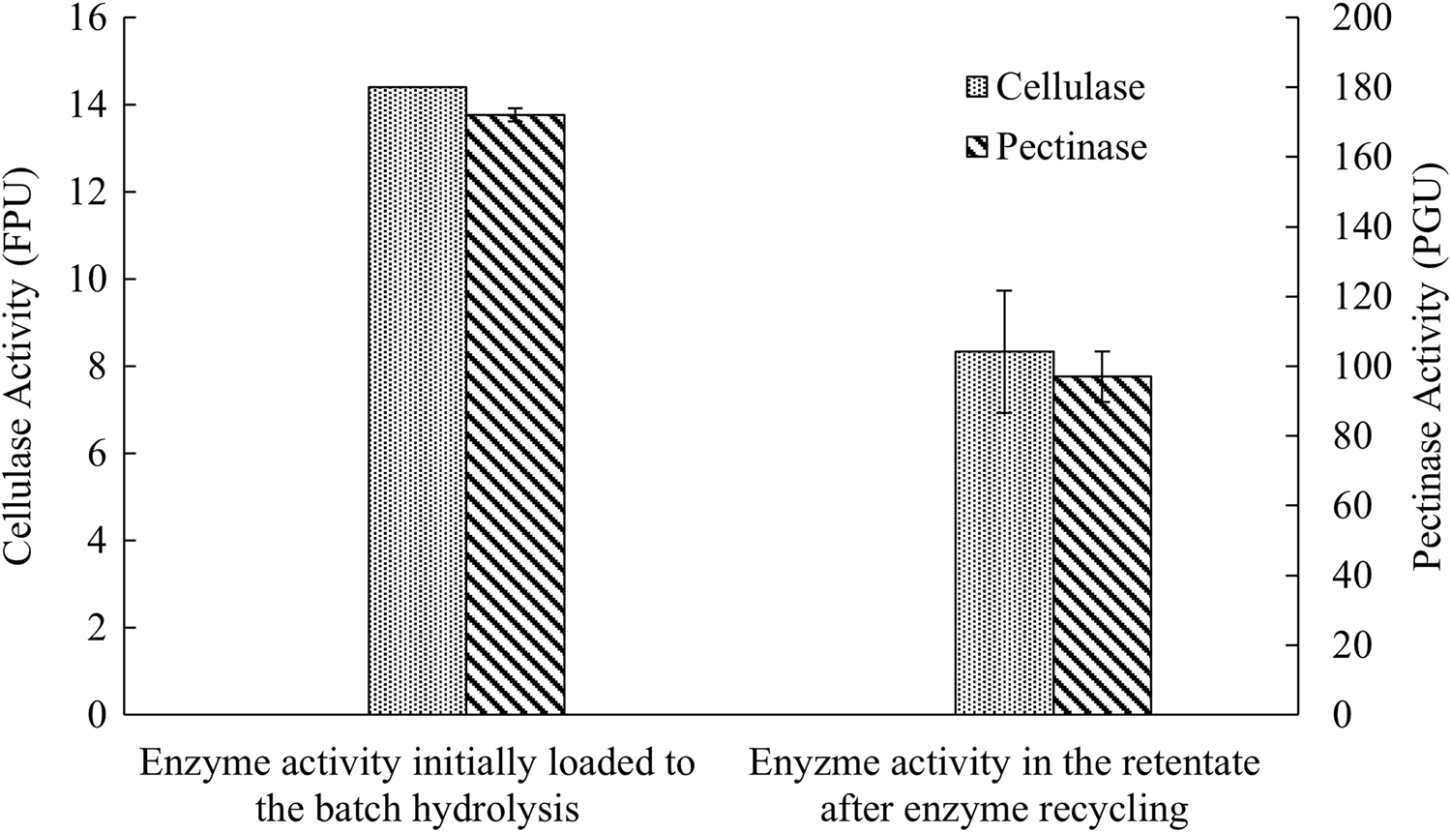
Cellulase and pectinase activities before and after enzyme recycling

Cellulase and pectinase activities in the retentate after centrifugation and ultrafiltration were measured by standard enzyme assays. As shown in Fig. 5, the cellulase and pectinase activities dropped from 14.4 to 8.3 FPU and 172.0–97.0 PGU, respectively, after the liquid recycling process. This represented a 42% and 44% reduction in cellulase and pectinase activities, respectively. Considering the standard deviation and potential enzyme denaturing after several runs of the recycling processes, additional 50% of fresh enzyme was concluded to be sufficient to subsequent batches of enzymatic hydrolysis of sugar beets.

### Enzymatic hydrolysis with solids recycling

**Fig. 6:**
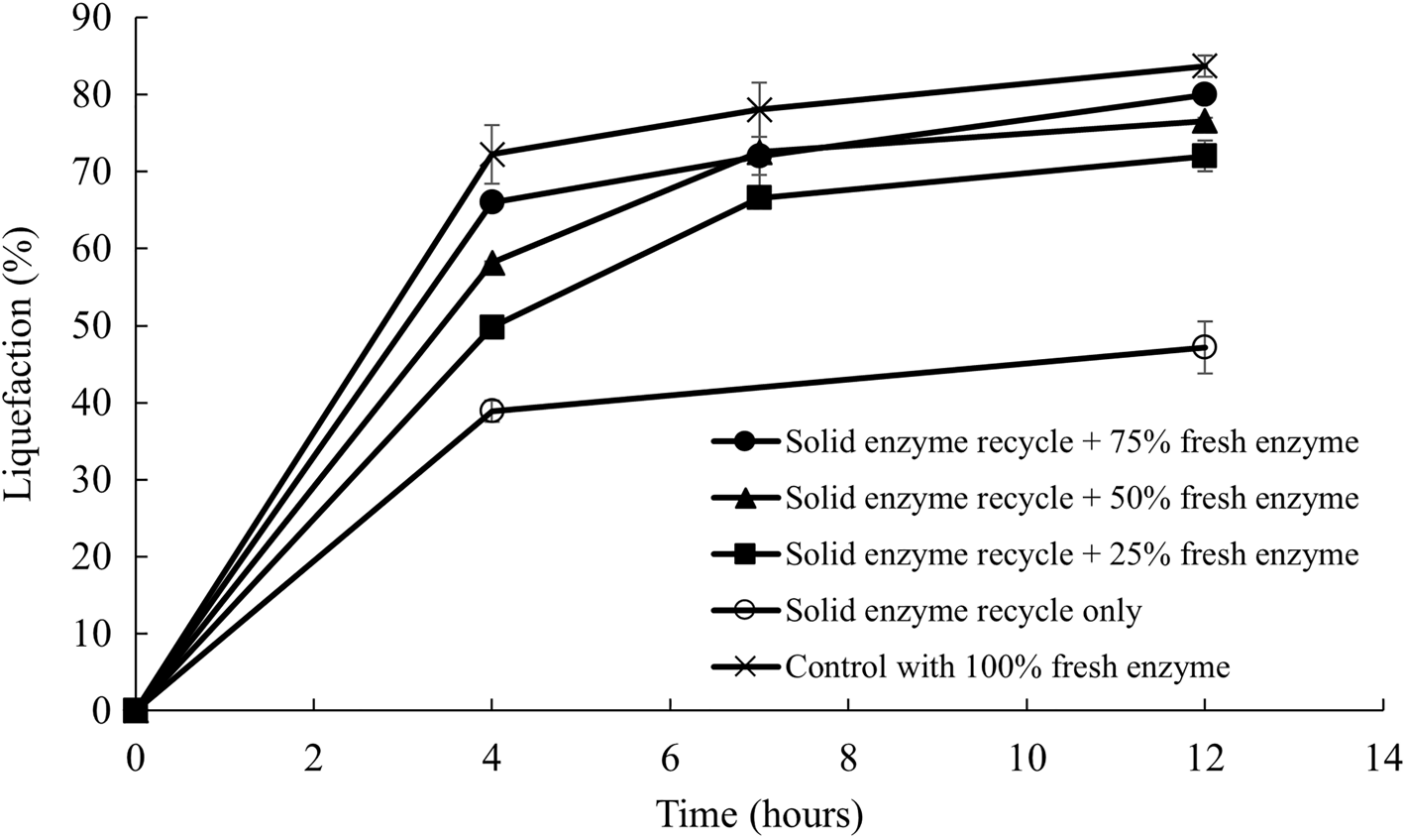
Liquefaction with recycled enzyme in the solid residue and fresh enzyme

Similar experiments were carried out to investigate the effect of solids recycling after centrifugation on the subsequent batch of hydrolysis of sugar beets. Like liquid recycling, different amounts of fresh enzymes were added to compensate for the enzyme losses. Figure 6 shows the liquefaction of sugar beets with solids recycling and different amounts of additional fresh enzymes during the hydrolysis. The negative control is not shown since no liquefaction was observed throughout the hydrolysis. The positive control with 100% fresh enzyme had the highest liquefaction of 84% after 12 h. Solids recycling with 75% fresh enzyme addition reached a 72% and 80% liquefaction extent after 8 and 12 h, respectively. Hydrolysis with less fresh enzyme addition had lower liquefaction rates and extents, meaning that at least 75% of fresh enzyme was needed to achieve the same liquefaction for a subsequent batch of sugar beet hydrolysis. This is reasonable because a portion of the enzymes could be irreversibly bound to the solids, more fresh enzymes, are therefore required to compensate for the enzyme losses.

**Fig. 7:**
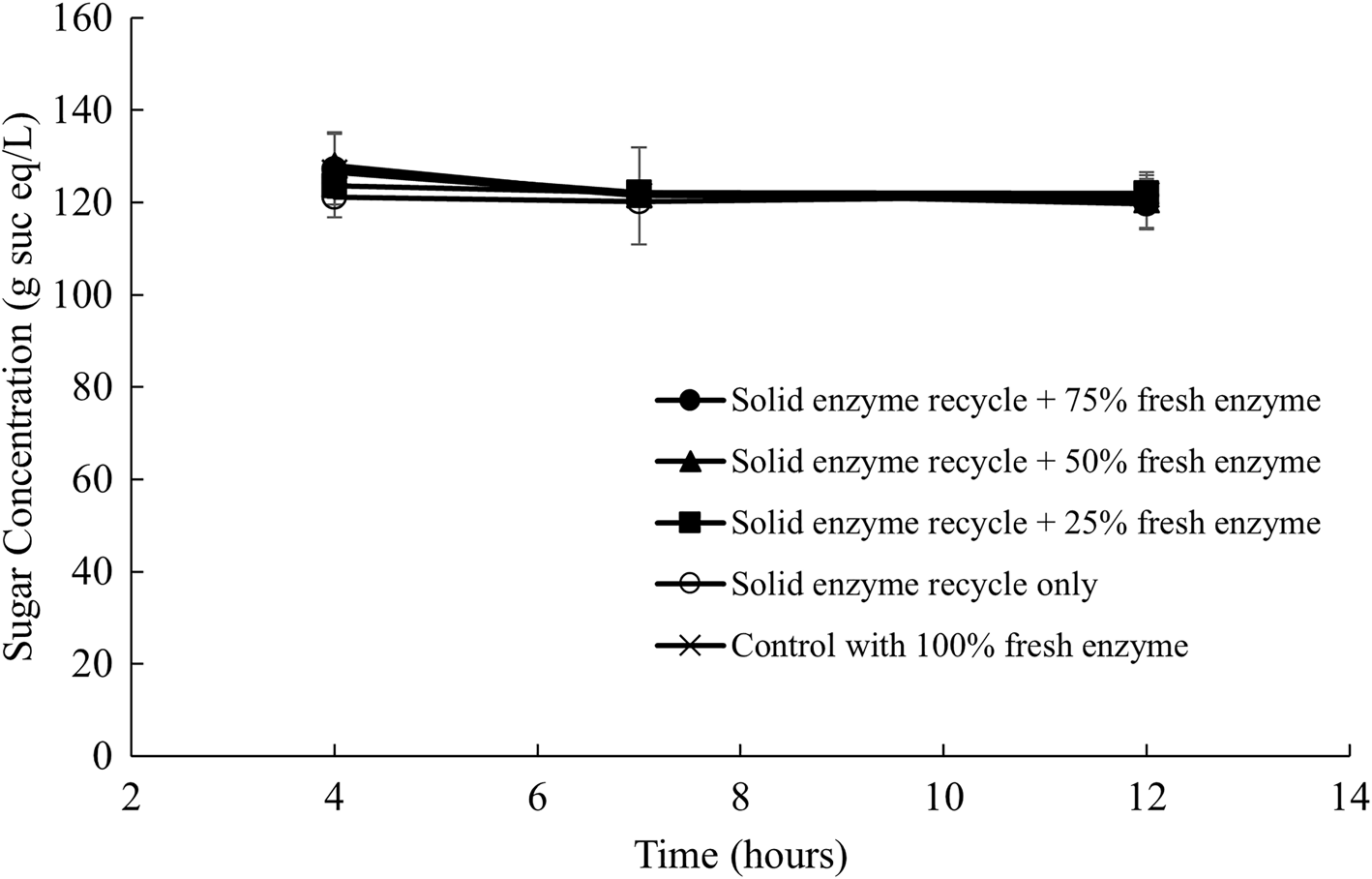
Sugar concentration in the supernatant with solids recycle and fresh enzyme

Sugar concentrations in the supernatant were also measured and shown in Fig. 7. Similar to liquid recycling, sugar concentration stayed constant at around 121 g sucrose eq/L during the enzymatic hydrolysis. Although the final sugar concentration was lower than what was observed in liquid recycling, it still accounts for around 89% of the initial sucrose in the beets since solids recycling reduced the mass of the fresh sugar beets loaded to the subsequent batch of hydrolysis by 20%. The differences of the sugar concentration between each treatment, as well as the positive control, were also smaller than those with liquid recycling. This could be the outcome that most of the soluble sugars were separated and suspended in the liquid stream during centrifugation.

**Fig. 8:**
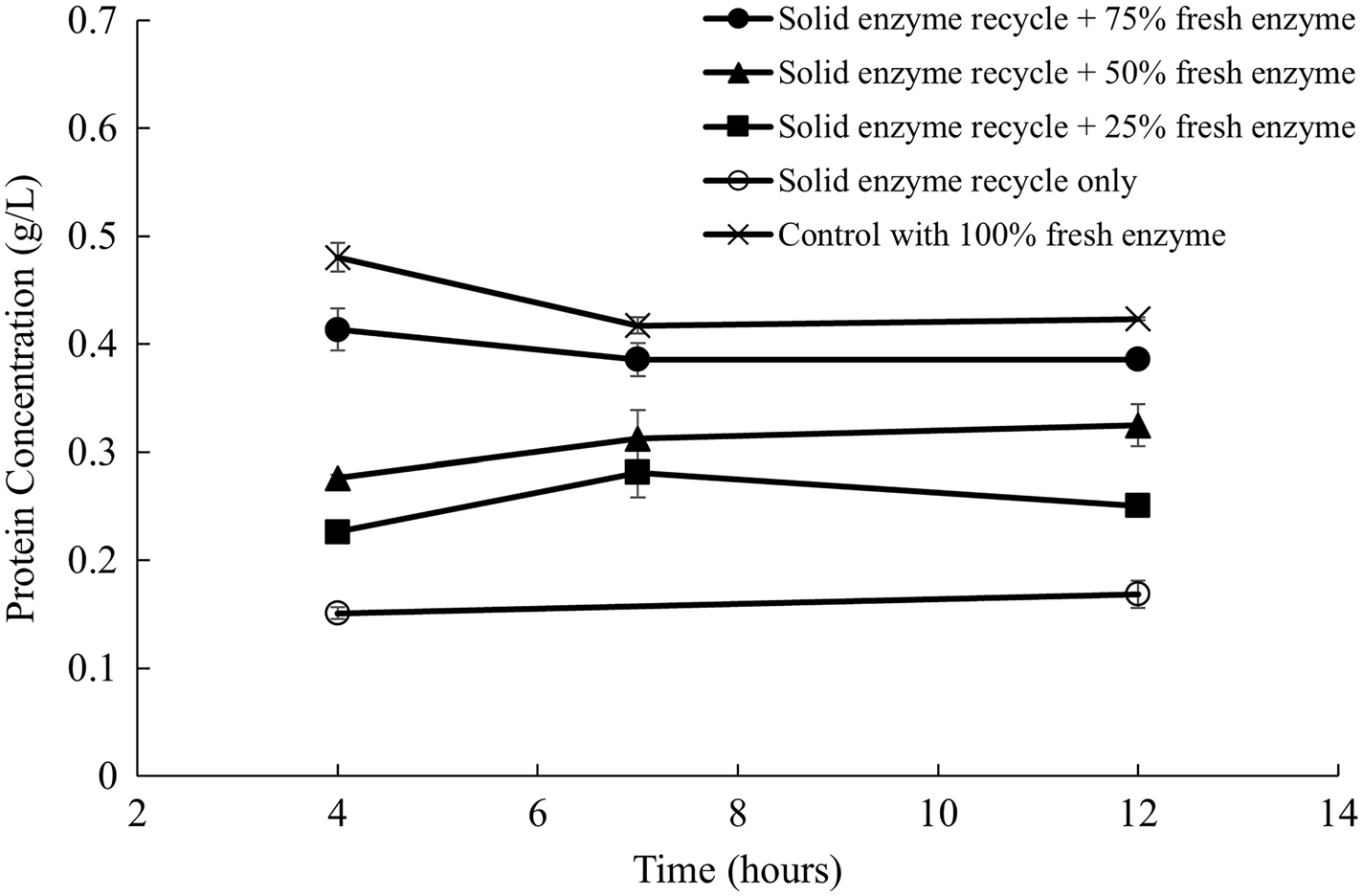
Protein concentration in the supernatant with solid recycling and fresh enzyme

The protein content of the positive control with fresh enzymes was around 0.44 g/L (Fig. 8), which is consistent with the result of the liquid recycling experiment shown in Fig. 4. The soluble protein concentration of the treatments with solid recycling and fresh enzyme addition was lower than that of the positive control. This could be an explanation for why the solids recycling showed lower efficiencies in liquefaction than the liquid recycling. The irreversible binding of enzymes to the solids could be accounted for the lower liquefaction extent, in which requires further research to trace the distribution of the enzymes during the hydrolysis of sugar beets. The mass balance of protein measurement was inconclusive because the Bradford Assay measures not only the enzyme protein, but also the soluble protein from sugar beets.

Measuring the enzyme activities in the solids after sugar beet hydrolysis would be challenging since various enzymes were bound to the surface of the solids while most of the laboratory enzyme assays could only accurately measure the enzyme activities in the liquid phase. Solids recycling showed a lower liquefaction efficiency than liquid recycling, as well as other issues, such as reducing the reactor capacity in the successive batches of soluble sugar production [11]. Due to these factors, only liquid recycling was considered in the later part of the research.

### Enzymatic hydrolysis with surfactant addition

Since around 50% of enzymes could be lost during liquid recycling processes and recycling the solids was concluded to be inefficient, surfactant was added during the enzymatic hydrolysis to help enzyme desorption from the binding sites on the surface of the solids to increase the efficiency of liquid recycling. As mentioned in the experimental procedures, surfactant was added to the beet at the beginning and in the middle of the enzymatic hydrolysis. Liquefaction, sugar concentration and enzyme activities were measured using the standard methods mentioned above.

**Fig. 9:**
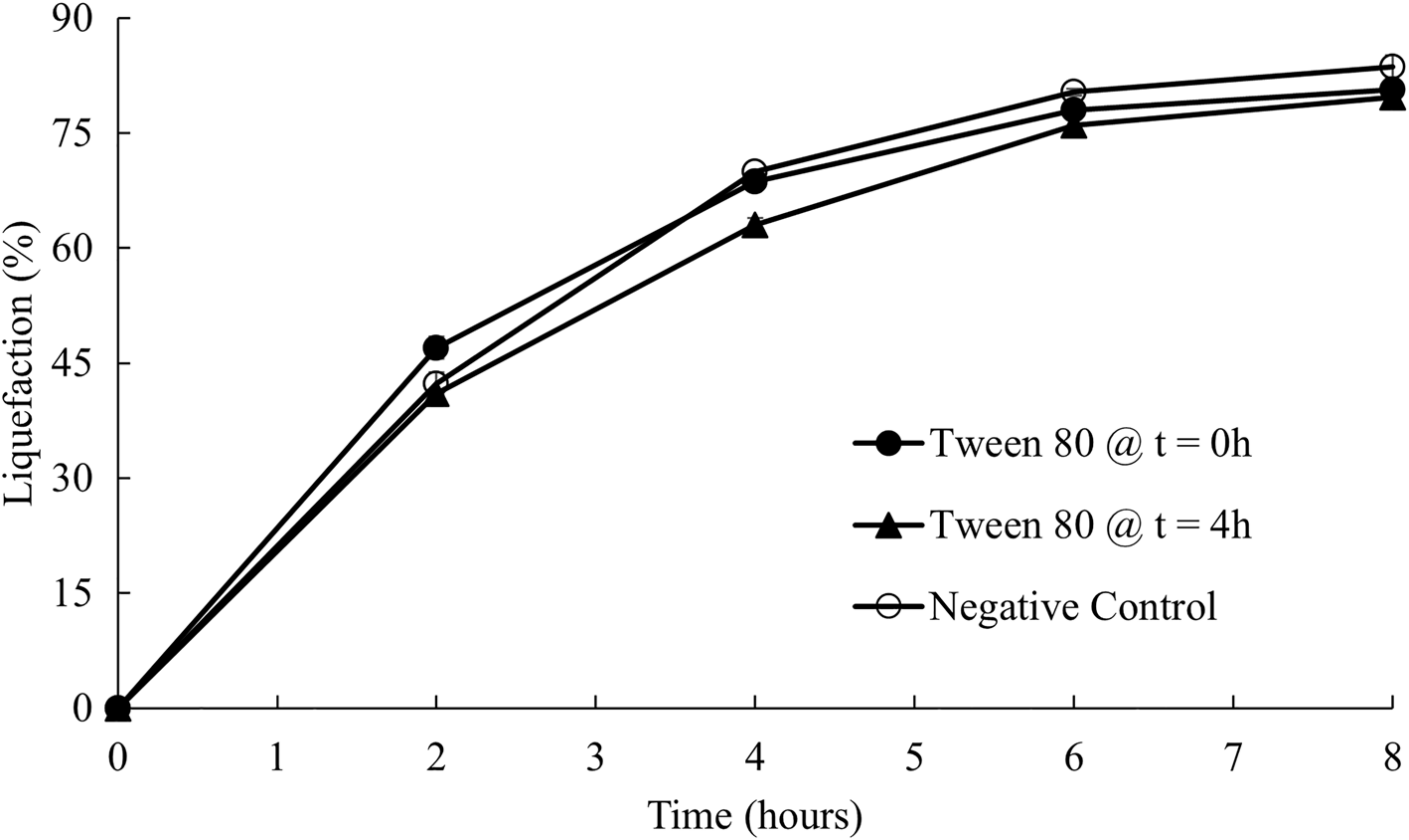
Enzymatic hydrolysis of sugar beets with surfactant addition

Figure 9 shows the liquefaction of surfactant addition at the beginning as well as in the middle (4 h) of the enzymatic hydrolysis of sugar beets. There was no significant difference in the liquefaction of treatments with surfactant addition compared to the control without surfactant. Therefore, adding Tween 80 as a surfactant was not shown to affect the liquefaction efficiency.

This conclusion agreed with a study on evaluating the effect of surfactant addition on the recovery of cellulase for corn stover hydrolysis [18]. However, it was contradictory to other research, which indicated that adding surfactant could not only increase the hydrolysis efficiency, but also reduce the enzyme dosage [19]. Helle et al. [20] found that adding surfactant could increase the cellulose hydrolysis efficiency by approximately seven times. The authors also concluded that surfactant addition could help cellulose structure disruption and reduce enzyme inactivation due to nonspecific adsorption onto the solids. The reasons why sugar beet hydrolysis with surfactant did not show significant effects could be that the cell wall structure of the sugar beet root is more accessible to the enzymes than that of the other lignocellulosic feedstocks. The lignin content, causing irreversible binding and enzyme inactivation, in the sugar beet root is also low as shown in Table 2.

**Table 2:** Lignin content in different biomass feedstocks [21, 22]

Feedstocks	Lignin (% dry basis)
Sugar beet	1.0
Corn stover	18.7
Oat straw	13.8
Rice straw	11.9
Wheat straw	16.0
Sugarcane bagasse	14.5

**Fig. 10:**
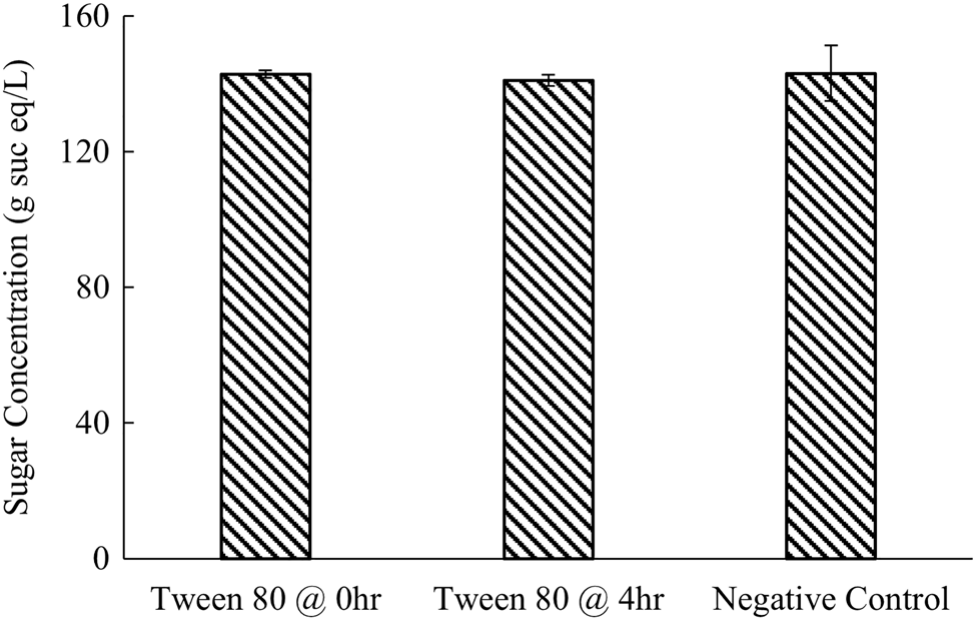
Sugar concentration in the supernatant after 8 h with surfactant addition

Similar to the liquefaction, final sugar concentrations with or without surfactant addition were consistent with the previous results as shown in Fig. 10. The final sugar concentration was 142.9, 141.1, and 143.1 g sucrose eq/L for surfactant addition at the beginning, in the middle, and negative control without surfactant, respectively, with no significant differences.

**Fig. 11:**
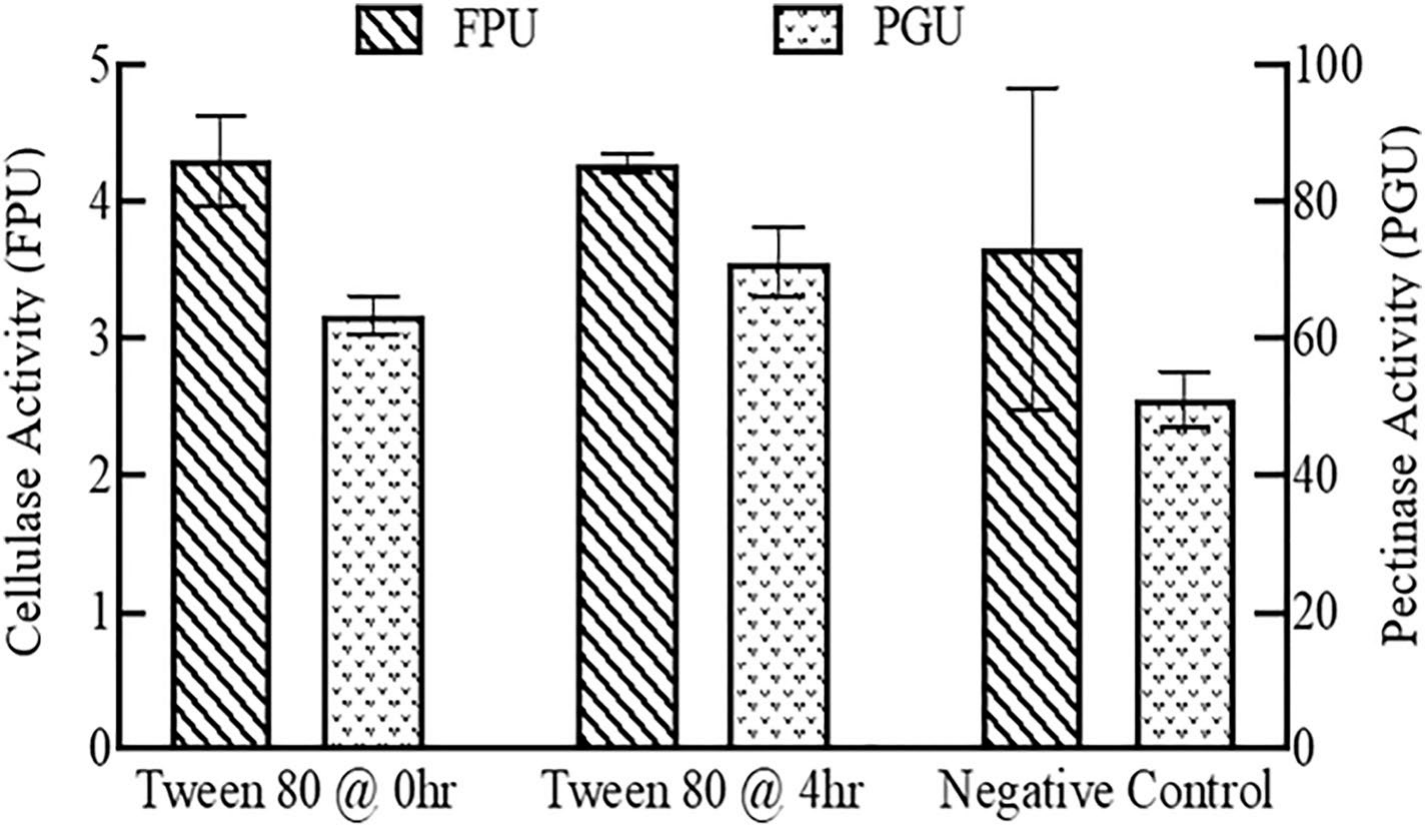
Cellulase and pectinase activities in the supernatant with surfactant addition

However, unlike the liquefaction and sugar concentration measurements, enzyme activities in the supernatant measured by standard enzyme activity assays were lower than those measured in the previous experiments. The cellulase activities in the hydrolysate were 4.31, 4.28, and 3.66 FPU for the surfactant addition at the beginning, middle, and negative control, respectively. The pectinase activities were 63.5, 71.2 and 51.18 PGU for the surfactant addition at the beginning, middle, and negative control, respectively. Although the enzyme activities between each treatment showed no significant difference, as shown in Fig. 11, they were lower than those measured in the hydrolysate in the enzyme recycling experiments.

Factors which contribute to the lower enzyme activities could include the treatments with Tween 80 which require more time for washing and recovery, resulting in the enzymes becoming denatured and inactivated during the rigorous ultrafiltration process. At least two cycles of ultrafiltration for 90 min at 5000×*g* was required to achieve the same purpose of washing out the soluble sugar using the centrifugal ultrafiltration concentrators before the enzyme activity measurements. Although some of the research suggested that surfactant brings positive impacts on enhancing the efficiency of enzymatic hydrolysis of lignocellulosic feedstock, adding surfactant to increase the liquid recycling efficiency may be inapplicable in the hydrolysis of sugar beets. The reduction may be due to the surfactant potentially forming another layer on the surface of the PES flat sheet membrane, which would increase the processing time and reduce the enzyme activity. Amirilargani et al. [23] observed the PES membrane thickness increased from 142 to 180 µm with Tween 80 addition. The membrane thickness is negatively proportional to the flux due to Eq. 5. Also, adding surfactant, such as Tween 80, would lower the hydrophile-lipophile balance (HLB) and increase the viscosity of the solution, which could cause the reduction of the permeate flux as well. Surfactant addition may be a more efficient approach with other enzyme recycling strategies, such as adding fresh substrate for enzyme readsorption. The basic theory of the reduction in permeate flux by adding surfactant is still under investigation; more research is needed to understand the intermolecular interaction between the surfactant and PES membrane surface.

### Sequential batches of enzymatic hydrolysis with enzyme recycling

**Fig. 12:**
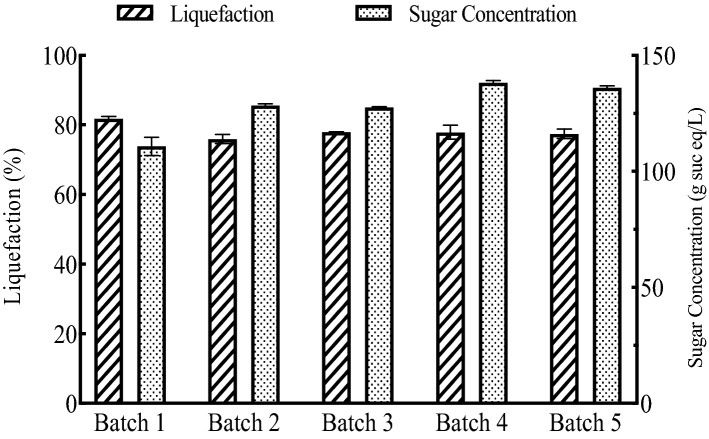
Liquefaction of sequential batches of enzymatic hydrolysis

To demonstrate sequential batches of hydrolysis with enzyme recycling as well as ensuring that no inhibitory effects would occur, five batches of hydrolysis with liquid recycling were conducted and both liquefaction and sugar concentrations were measured. Figure 12 shows the liquefaction and final sugar concentration in the supernatant of five sequential batches of enzymatic hydrolysis. There was no significant difference in liquefaction between each batch of hydrolysis. The initial batch with 100% fresh enzymes showed a lower sugar content than the remaining four batches with liquid recycling, which is due to the soluble sugar contained in the retentate with the recycled enzymes. The later four batches of sugar beet hydrolysis had similar performances, meaning that at least five sequential batches of hydrolysis could be successfully conducted with liquid recycling and without compromising either the quantity nor quality of the final product.

The final sugar production was calculated by multiplying the final liquid volume and sugar concentration. The yield was the mass fraction of the total soluble sugar to the dry weight of the initial sugar beet. Table 3 summarizes the final sugar production and yields of five sequential batches of enzymatic hydrolysis with enzyme recycling. It is promising that at least five batches could be successfully conducted by applying the enzyme recycling strategy. The average sugar yield of five batches of hydrolysis was 50%, which is approximately 22% lower than the theoretical sucrose content in the dry sugar beet root. The 22% sugar loss could have ended up in the solid residue as an outcome of the solid liquid separation.

**Table 3:** Sugar production and yields of sequential batches of hydrolysis

Batch	Sugar Production (g sucrose eq)	Yield (% dry basis)
1	1.36 ± 0.05	45 ± 1.7
2	1.46 ± 0.03	49 ± 1.0
3	1.49 ± 0.00	50 ± 0.0
4	1.62 ± 0.04	54 ± 1.3
5	1.58 ± 0.03	53 ± 1.0

## Conclusions

The process design and potential application of enzyme recycling during enzymatic hydrolysis of sugar beets was presented in this article. Centrifugation and ultrafiltration were applied in the process to separate and recover the enzymes, which were reused in the subsequent batches of beet hydrolysis. Both 10 and 50 kDa MWCO PES ultrafiltration membranes were found to be capable of separating and retaining a majority of the enzymes in the retentate with no significant fouling within 2 h of continuous operation. However, 50 kDa MWCO membrane was more efficient in terms of the permeate flux, thus, it was examined in the laboratory scale enzyme recycling experiments.

By adding the retentate, containing approximately 50% of the original enzyme activities, with an additional 50% of the fresh enzymes, the subsequent batch of enzymatic hydrolysis achieved a similar liquefaction compared to the first batch. Additionally, up to five sequential batches of enzymatic hydrolysis with liquid recycling were successfully conducted with comparable liquefaction extent and sugar yields. Therefore, enzyme recycling using centrifugation and ultrafiltration could be a promising way to reduce the enzyme usage and make the overall sugar beet soluble sugar production process more economically feasible. The effect of solid recycling on the hydrolysis of sugar beets was also investigated by adding the solids separated by centrifugation to a subsequent batch of hydrolysis with varying amounts of fresh enzymes. However, solid recycling was less efficient than liquid recycling, and might cause problems in material handling and reduce the reactor’s effective volume. Therefore, solids after the hydrolysis were not recommended to be recycled using this enzyme recycling strategy. Furthermore, Tween 80 was added during the enzymatic hydrolysis to investigate the effect of surfactant addition on the hydrolysis efficiency with liquid recycling. Liquefaction extent with Tween 80 addition showed no significant difference between the control without any surfactant. Adding surfactant, on the other hand, significantly reduced the efficiency of the membrane ultrafiltration.

To enhance the economic feasibility of enzymatic hydrolysis of sugar beets, the efficiency of enzyme recycling needs be further improved. This could be achieved by modifying the membrane structure to selectively retain useful enzymes or accelerating the enzyme desorption process. Additionally, finding a new way to valorize the residual solids could also be an efficient approach to increase the revenue from the side stream.
